# BCI-Utility Metric for Asynchronous P300 Brain-Computer Interface Systems

**DOI:** 10.1109/TNSRE.2023.3322125

**Published:** 2023-10-16

**Authors:** Guoxuan Ma, Jian Kang, David E. Thompson, Jane E. Huggins

**Affiliations:** Department of Biostatistics, University of Michigan, Ann Arbor, MI 48109 USA; Department of Biostatistics, University of Michigan, Ann Arbor, MI 48109 USA; Department of Electrical and Computer Engineering, Kansas State University, Manhattan, KS 66506 USA; Department of Biostatistics, University of Michigan, Ann Arbor, MI 48109 USA; Physical Medicine and Rehabilitation Department, Michigan Medicine, and the Department of Biomedical Engineering, College of Engineering, University of Michigan, Ann Arbor, MI 48109 USA

**Keywords:** Brain–computer interface (BCI), BCI performance metrics, ERP BCI speller

## Abstract

The Brain-Computer Interface (BCI) was envisioned as an assistive technology option for people with severe movement impairments. The traditional synchronous event-related potential (ERP) BCI design uses a fixed communication speed and is vulnerable to variations in attention. Recent ERP BCI designs have added asynchronous features, including abstention and dynamic stopping, but it remains a open question of how to evaluate asynchronous BCI performance. In this work, we build on the BCI-Utility metric to create the first evaluation metric with special consideration of the asynchronous features of self-paced BCIs. This metric considers accuracy as all of the following three – probability of a correct selection when a selection was intended, probability of making a selection when a selection was intended, and probability of an abstention when an abstention was intended. Further, it considers the average time required for a selection when using dynamic stopping and the proportion of intended selections versus abstentions. We establish the validity of the derived metric via extensive simulations, and illustrate and discuss its practical usage on real-world BCI data. We describe the relative contribution of different inputs with plots of BCI-Utility curves under different parameter settings. Generally, the BCI-Utility metric increases as any of the accuracy values increase and decreases as the expected time for an intended selection increases. Furthermore, in many situations, we find shortening the expected time of an intended selection is the most effective way to improve the BCI-Utility, which necessitates the advancement of asynchronous BCI systems capable of accurate abstention and dynamic stopping.

## I. Introduction

A BRAIN-COMPUTER interface (BCI) is designed for the direct operation of external technology without physical movement using brain signals. While BCIs were originally envisaged as an assistive technology option for people with severe motion impairments, the applications of BCIs now encompass rehabilitation treatments, recreational applications, and passive monitoring of electroencephalogram (EEG) responses [1], [2].

The event-related potential (ERP) BCI design [3] presents multiple stimuli in an on-screen keyboard. The user then selects stimuli one at a time. The stimulus the user is currently trying to select is called the target stimulus. The BCI classifies the EEG response after each stimulus as a target or non-target according to whether it contains the ERPs that are induced when the user perceives a target stimulus. This ERP BCI design is often named after the largest component of these ERPs, the P300, which is a positive deflection in the EEG signal that peaks about 300 ms after the onset of the rare and unexpected target stimulus [4], [5]. Among the many applications of P300-based BCIs, the P300 speller is a BCI typewriter that types characters from a virtual keyboard by flashing groups of characters randomly [3].

The original P300 BCI design [3] has a synchronous nature. The synchronous (system-paced) BCI design assumes that the user is always trying to control the BCI to make selections. It presents a fixed number of sequences of stimuli in a trial and always makes a selection after the trial ends, regardless of whether the user is actually paying attention to the BCI. As a result, the synchronous design is vulnerable to variations in attention and does not maximize the communication speed. Recent ERP BCI designs have added asynchronous (self-paced) features, including abstention and dynamic stopping [6], [7], [8], [9], [10], [11], [12], [13]. Abstention refers to the functionality of a BCI system that skips a trial or declines to make any selection when the selection is a potential error. On the other hand, dynamic stopping refers to the capability of producing a selection as soon as the information from the sequences that have been presented are calculated to be adequate for an accurate selection [6], [14], [15]. Either abstaining from producing a selection or producing a selection quickly should result in improvements in BCI usability and an asynchronous BCI selection rate.

System performance evaluation is a vital step for BCI system development. Many performance metrics have been used to evaluate the communication capability of a BCI system, including classification accuracy, Cohen’s Kappa coefficient, confusion matrix, mutual information, information transfer rate, etc [16], [17]. Some metrics have been used as gold standards in other communication tasks, but they have limitations in evaluating BCI system performance. Classification accuracy, Cohen’s Kappa coefficient and the confusion matrix do not account for the time needed for making a selection and thus do not measure throughput of a BCI system. Mutual information requires the estimation of the joint statistical distribution of the system input and output, which is often impractical in BCI research since the number of selections in a BCI study is typically small [16]. While information transfer rate combines accuracy and speed into a single metric, it only provides an unrealistic theoretical upper bound of bits transferred for a BCI system and cannot incorporate error correction and other rate enhancements [18], [19].

The BCI-Utility metric [18] is a user-centered metric specifically designed for BCI system evaluation. It attempts to measure the average benefit with a BCI system over trials and is maximized when the most benefit is obtained in the shortest interval of time. Dal Seno et al. presented the BCI-Utility formula for a P300 Speller with and without error correction. However, the BCI-Utility metric is highly dependent on the system and experimental design, and thus interface-specific. The formulas derived by Dal Seno et al. have been sufficient for most applications in evaluating fully-synchronous P300 spellers [20], [21], [22], [23], so only a few works have derived new BCI-Utility metric formulas for new cases [24], [25], [26], [27], [28].

Most metrics for BCI performance evaluation, including the BCI-Utility metric, predate the design concepts of asynchronous ERP BCI – abstention and dynamic stopping. Metric development for asynchronous BCIs is more challenging than that of synchronous BCIs. The overall throughput, which is a key metric for synchronous BCIs, may not be proper for asynchronous BCIs incorporating idle periods [16]. The complexity of asynchronous BCI systems requires a metric that accounts for both accuracy and efficiency over active periods, and correct detection of inaction over idle periods. However, to our knowledge, there is no literature on the development of metrics for evaluating asynchronous BCI system performance. Therefore, in this paper, we present the BCI-Utility metric for asynchronous P300 BCI spellers. We provide the derivation in the context of the P300 speller, but the derived metric can be easily applied to evaluate other asynchronous BCIs with slight or even without alterations.

The rest of the paper is structured as follows. Section II first revisits the BCI-Utility metric for a fully-synchronous P300 speller and then derives new formulas for the BCI-Utility metric for three cases of P300 BCI spellers with asynchronous features. Then, we validate our derived BCI-Utility metric by extensive simulations mimicking the realistic use of the asynchronous P300 spellers in Section III. In Section IV, we provide and describe BCI-Utility curves under different scenarios of parameter settings. We illustrate and discuss the practical usage of the derived metric on real-world BCI performance data in Section V, and conclude our paper in Section VI with a discussion of limitations of our work.

## II. BCI-Utility

The BCI-Utility [18] is defined as

(1) $$U=E\left[\underset{T\to \infty }{\text{lim}}\frac{\int_{0}^{T}b\text{(}t\text{)}dt}{T}\right]$$

where b(t) is a non-negative function that measures users’ satisfaction with the BCI system at time t. For a discrete BCI where the output is defined only at discrete time instants, the benefit function is defined only at time instant $t_{k}$ when the output is generated. Therefore, the benefit function in (1) becomes

$$b\text{(}t\text{)}=\sum_{k=1}^{K}b_{k}\delta \text{(}t-t_{k}\text{)}$$

where $b_{k}$ is the benefit received at $t_{k}$ and K is the number of output, in time interval [0,T], and δ(·) is the Dirac delta function. Let $\Delta t_{k}=t_{k}-t_{k-1}$ for all k=1,2,… and define $t_{0}=0$, we have $T={\text{Σ}}_{k=1}^{K}\text{Δ}t_{k}$. Then, (1) becomes

(2) $$U=E\left[\underset{T\to \infty }{\text{lim}}\frac{{\text{Σ}}_{k=1}^{K}\int_{0}^{T}b_{k}\delta \left(t-t_{k}\right)dt}{T}\right]\;\;=E\left[\underset{K\to \infty }{\text{lim}}\frac{{\text{Σ}}_{k=1}^{K}b_{k}}{{\text{Σ}}_{k=1}^{K}\text{Δ}t_{k}}\right]\;\;=E\left[\underset{K\to \infty }{\text{lim}}U_{K}\right]$$

where we define $U_{K}=\sum_{k=1}^{K}b_{k}\text{/}\sum_{k=1}^{K}\text{Δ}t_{k}$ as the average benefit over the first K outputs.

In this section, we discuss BCI-Utility under four scenarios, each with its own subsection (II-A–II-D). As the first scenario, we revisit the BCI-Utility of a fully-synchronous P300 speller discussed by Dal Seno et al. [18] in Section II-A. Then, in Section II-B, we extend the first scenario by incorporating dynamic stopping where a trial could terminate once sufficient evidence are collected for making a decision before all sequences are completed. In the third case, we allow for the abstention of the P300 speller with dynamic stopping in Section II-C. Finally in Section II-D, we discuss the BCI-Utility of a P300 speller with abstention and dynamic stopping when a skip can be intentional.

### A. BCI-Utility for a Plain P300 Speller [18]

In this section, we revisit an explicit formula for the BCI-Utility metric for a fully-synchronous P300 speller by Dal Seno et al. [18]. A fully-synchronous P300 speller is designed in a simple way. The BCI makes a selection and displays the selection on the screen at the end of each trial. If the selection is correct, the user moves on to the next selection; otherwise, the user needs to type a backspace to delete the incorrect selection. The speller has N possible outcomes for all trials, with N−1 selections and a backspace. Following [18], we assume

A1 The accuracy of the system is constant over the trials, thus no time-dependency is included in the model.

A2 The system is memoryless, thus each trial is not influenced by the result of the previous ones.

By the above assumptions, both $\left\{b_{k}\right\}$ and $\left\{\Delta t_{k}\right\}$ are ergodic processes. For a random variable X generated by an ergodic process $\left\{X_{k}\right\}$, we have

$$\underset{K\to \infty }{\text{lim}}\frac{\sum_{k=1}^{K}X_{k}}{K}=E\text{[}X\text{]}.$$

Therefore, (2) reduces to

(3) $$U=E\left[\frac{E\text{[}b\text{]}}{E\text{[Δ}t\text{]}}\right]=\frac{E\text{[}b\text{]}}{E\text{[Δ}t\text{]}}=\frac{b_{sel}}{T_{sel}}$$

where $b_{sel}$ is the expected benefits of a correct selection and $T_{sel}$ is the expected time needed to make a correct selection. We could set $b_{sel}=1$ to assign unit benefit to any selection, or measure the benefit by the conveyed information $b_{sel}={\text{log}}_{2}\text{(}N-1\text{)}$ assuming equal probability among selections.

For the purpose of computing $T_{sel}$, we denote $c_{T}$ as the time needed to complete all sequences in a trial, p as the accuracy of the speller and $T_{bs}$ as the time needed to type a backspace. We have two possible cases in each trial,

- The P300 speller makes the correct selection. This happens with probability p, and it takes $c_{T}$ to complete all sequences in the trial for making such a selection.
- The P300 speller makes the wrong selection. This happens with probability 1−p, and it takes $c_{T}$ to make the wrong selection, $T_{bs}$ to delete the wrong selection and $T_{sel}$ to select the correct one.

Then, the expected time for a correct selection is

(4) $$\begin{array}{l} T_{sel}=pc_{T}+\text{(}1-p\text{)}\;\text{(}c_{T}+T_{bs}+T_{sel}\text{)}\\ \;\;=c_{T}+\text{(}1-p\text{)}\;\text{(}T_{bs}+T_{sel}\text{)}. \end{array}$$

Similarly, the expected time for a backspace is

(5) $$T_{bs}=pc_{T}+\text{(}1-p\text{)}\;\text{(}c_{T}+T_{bs}+T_{bs}\text{)}.$$

By subtracting (4) and (5) we get

$$p\left(T_{sel}-T_{bs}\right)=0\text{,}$$

thus $T_{sel}=T_{bs}$ provided p>0. Then, from (4) we have

$$T_{sel}=c_{T}+\text{(}1-p\text{)}\;\text{(}2T_{sel}\text{)}.$$

Therefore,

(6) $$T_{sel}=\frac{c_{T}}{2p-1}$$

where we require p>0.5 . If p≤0.5, the expected time for a correct selection $T_{sel}$ will diverge to infinity. Plugging $b_{sel}={\text{log}}_{2}\text{(}N-1\text{)}$ and (6) into (3) we get

(7) $$U=\left\{\begin{array}{ll} \frac{\text{(}2p-1\text{)}\;{\text{log}}_{2}\text{(}N-1\text{)}}{c_{T}}\text{,} & p>0.5\\ 0\text{,} & p\leq 0.5. \end{array}\right.$$

### B. BCI-Utility in a P300 Speller With Dynamic Stopping

We now consider a P300 speller with dynamic stopping. The design of the speller is similar to that described in Section II-A except now the BCI can make a selection or type a backspace before all sequences are completed in a trial. When the speller collects sufficient information before all sequences are ended in a trial, the speller will make a decision, end the trial early and move on to the next trial. We consider the same assumptions in Section II-A and thus (3) holds in this case. We introduce $0<c\leq c_{T}$ as the expected time for a trial and replace $c_{T}$ with c in the two cases described in Section II-A. The expected time for a correction selection and the expected time for a backspace become

$$T_{sel}=pc+\text{(}1-p\text{)}\;\text{(}c+T_{bs}+T_{sel}\text{)}$$

$$T_{bs}=pc+\text{(}1-p\text{)}\;\text{(}c+T_{bs}+T_{bs}\text{)}$$

and with the same argument in Section II-A we get

(8) $$U=\left\{\begin{array}{ll} \frac{\text{(}2p-1\text{)}\;{\text{log}}_{2}\text{(}N-1\text{)}}{c}\text{,} & p>0.5\\ 0\text{,} & p\leq 0.5. \end{array}\right.$$

Note that the BCI-Utility for a plain P300 speller in (7) is special case of (8) for which $c=c_{T}$. If we disable the dynamic stopping functionality of the BCI system, then $c=c_{T}$ and we obtain the same formula from (8) as the BCI-Utility in Section II-A.

### C. BCI-Utility in a P300 Speller With Abstention and Dynamic Stopping

In this section, we derive the BCI-Utility for a P300 speller with abstention and dynamic stopping. During each trial, the BCI will make a selection or type a backspace, end the trial and move on to the next trial once there is sufficient evidence. If all sequences are completed but no decision is made due to lack of evidence, the speller will abstain and skip the trial without making any selection or typing backspace. Note, in this section, abstention serves only to avoid poorly-evidenced selections. Intentional abstentions will be discussed in the next section. We extend assumption A1 in Section II-A,

A1a The selection accuracy of the system and the probability of abstention are constant over the trials, thus no time-dependency is included in the model.

With assumption A1a and other assumptions described in Section II-A, (3) holds for a P300 speller with abstention and dynamic stopping.

For the purpose of computing $T_{sel}$, we first introduce some notation,

- $p^{sel}$: the probability of making a selection
- $p^{skip}=1-p^{sel}$: the probability of skipping a trial without typing either letters or backspace
- $p_{sel}^{correct}$: the probability of correct selection given a selection being made
- $p_{sel}^{wrong}=1-p_{sel}^{correct}$: the probability of incorrect selection given a selection being made
- $c_{T}$: the duration of a complete trial
- c: the expected duration of a trial; $c<c_{T}$ if a decision is made before all sequences are completed
- $T_{bs}$: the expected time of typing a backspace.

Then for each trial, we have three possible cases,

- A selection is made and the selection is correct. This happens with probability $p^{sel}p_{sel}^{correct}$, and c is the time needed to make the selection.
- A selection is made but the selection is wrong. This happens with probability $p^{sel}p_{sel}^{wrong}$, and it takes c to spell the wrong letter, and extra $T_{bs}+T_{sel}$ to delete the wrong selection and make the right one.
- The algorithm decides to skip the trial without typing any letters or backspace. This happens with probability $p^{skip}$. It takes $c_{T}$ to end the trial and $T_{sel}$ to type the letter.

Therefore, the expected time to spell a letter $T_{sel}$ is

(9) $$T_{sel}=p^{sel}p_{sel}^{correct}c+p^{sel}p_{sel}^{wrong}\left(c+T_{bs}+T_{sel}\right)+p^{skip}\left(c_{T}+T_{sel}\right)$$

Similarly, the expected time to type a backspace $T_{bs}$ is

(10) $$T_{bs}=p^{sel}p_{sel}^{correct}c+p^{sel}p_{sel}^{wrong}\left(c+T_{bs}+T_{bs}\right)+p^{skip}\left(c_{T}+T_{bs}\right)$$

and from (10) and (9) we obtain $T_{sel}=T_{bs}$. Then, $T_{sel}$ can be written as

$$T_{sel}=p^{sel}c+\left(1-p^{sel}\right)c_{T}+\left(1+p^{sel}-2p^{sel}p_{sel}^{correct}\right)T_{sel}$$

which implies

(11) $$T_{sel}=\frac{p^{sel}c+\left(1-p^{sel}\right)c_{T}}{p^{sel}\left(2p_{sel}^{correct}-1\right)}$$

for $p_{sel}^{correct}>0.5$ and $p^{sel}>0$. When $p_{sel}^{correct}\leq 0.5$ or $p^{sel}=0$, the expected time $T_{sel}$ to make an intended selection diverges to infinity. With $b_{sel}={\text{log}}_{2}\text{(}N-1\text{)}$ and (11), we finally get

(12) $$U=\left\{\begin{array}{ll} \frac{{\text{log}}_{2}(N-1)}{T_{sel}}\text{,} & p_{sel}^{correct}>0.5\;\text{and}\;p^{sel}>0\\ 0\text{,} & p_{sel}^{correct}\leq 0.5\;\text{or}\;p^{sel}=0 \end{array}\right.$$

where $T_{sel}$ is as in (11).

Note that (7) in Section II-A and (8) in Section II-B are special cases of (12). We have already shown the BCI-Utility in Section II-B is the same as that in Section II-A when dynamic stopping is disabled. When there is no abstention as in Section II-B, $p^{sel}=1$ and $p^{skip}=0$, and $p_{sel}^{correct}$ in this section has the same meaning as p in Section II-B. Therefore, in this case, (8) reduces to (12).

### D. BCI-Utility in a P300 Speller With Intentional Skip, Abstention and Dynamic Stopping

Here, We discuss the BCI-Utility for a P300 speller with abstention and dynamic stopping when a trial skip can be intentional. The design of the P300 speller is identical to that in Section II-C. However, now the intended outcomes not only include available selections and but may also include a skip of a trial. We model the process of benefits $\text{\{}b_{k}\text{\}}$ as a mixture of two processes of benefits $\text{\{}b_{k\text{,}sel}\text{\}}$ and $\text{\{}b_{k\text{,}skip}\text{\}}$,

$$b_{k}=s_{k}b_{k\text{,}sel}+\left(1-s_{k}\right)b_{k\text{,}skip}\;s_{k}∼\text{Bernoulli}\left(\pi_{k\text{,}sel}^{i}\right)$$

where $\text{\{}b_{k\text{,}sel}\text{\}}$ is the process of benefits for intended selections and $\text{\{}b_{k\text{,}skip}\text{\}}$ is the process of benefits for intentional skips; $s_{k}$ is a binary process indicating the membership in the mixture; $\pi_{k\text{,}sel}^{i}$ is the probability of intentional selections at time instant k. We further extend assumption A1a to A1b

A1b The selection accuracy of the system, the probability of selections being intended, and the probability of system abstention given user’s intention are constant over time, thus no time-dependency is included in the model.

The assumption that the probability of selections being intended is constant implies the process $s_{k}$ is a Bernoulli process consisting of i.i.d. random variables $\text{\{}s_{k}\text{\}}$ with $\pi_{k\text{,}sel}^{i}=\pi_{sel}^{i}$. Then, $\text{\{}b_{k}\text{\}}$ is an ergodic process. Similarly, we model the process $\text{\{}\Delta t_{k}\text{\}}$ as a mixture of $\text{\{}\Delta t_{k\text{,}sel}\text{\}}$ and $\text{\{}\Delta t_{k\text{,}skip}\text{\}}$,

$$\text{Δ}t_{k}=s_{k}\text{Δ}t_{k\text{,}sel}+\left(1-s_{k}\right)\text{Δ}t_{k\text{,}skip}\;s_{k}∼\text{Bernoulli}\left(\pi_{sel}^{i}\right)$$

and $\text{\{}t_{k}\text{\}}$ is an ergodic process. Then, from (2) we have

(13) $$U=E\left[\frac{E\text{[}b\text{]}}{E\text{[Δ}t\text{]}}\right]\;\;=E\left[\frac{\pi_{sel}^{i}b_{sel}+\pi_{skip}^{i}b_{skip}}{\pi_{sel}^{i}T_{sel}+\pi_{skip}^{i}T_{skip}}\right]\;\;=\frac{\pi_{sel}^{i}b_{sel}+\pi_{skip}^{i}b_{skip}}{\pi_{sel}^{i}T_{sel}+\pi_{skip}^{i}T_{skip}}$$

where we denote $b_{sel}$ and $b_{skip}$ as the expected benefit carried by any correctly spelled letter and the benefit carried by a correct skip, and $T_{sel}$ and $T_{skip}$ as the expected time to type an intended letter and the expected time to make an intentional skip. The benefits $b_{sel}$ and $b_{skip}$ can be customized values. Assuming equal probability among selections, the conveyed information is $b_{sel}={\text{log}}_{2}\text{(}N-1\text{)}$ bits.

For the purpose of computing $T_{sel}$ and $T_{skip}$, we first introduce some notation,

- $p_{skip}^{sel}$: the probability of the system making a selection given a skip is intended
- $p_{skip}^{skip}=1-p_{skip}^{sel}$: the probability of the system skipping a trial given a skip is intended
- $p_{sel}^{sel}$: the probability of the system making a selection given a selection is intended
- $p_{sel}^{skip}=1-p_{sel}^{sel}$: the probability of the system skipping a trial given a selection is intended
- $p_{sel}^{correct}$: the probability of correct selection given a selection being intentional and being made
- $p_{sel}^{wrong}$: the probability of incorrect selection given a selection being intentional and being made
- $c_{T}$: the duration of a complete trial
- c: the expected duration of a trial; $c<c_{T}$ if a decision is made before all sequences are completed
- $T_{bs}$: the expected time of typing a backspace.

Then for a trial where a skip is intended, we have two possible cases,

- The system makes a selection. This happens with probability $p_{skip}^{sel}$ and it takes $c+T_{bs}$ to make the selection and delete the selection and then $T_{skip}$ to make the intended skip.
- The system makes the right decision to skip. This happens with probability $p_{skip}^{skip}$ and it takes $c_{T}$ to make the decision to skip.

Therefore, the expected time for an intended skip $T_{skip}$ is

(14) $$T_{skip}=p_{skip}^{sel}\left(c+T_{bs}+T_{skip}\right)+p_{skip}^{skip}c_{T}.$$

For a trial where a selection is intended, we have three possible cases,

- The system makes a selection as intended. This happens with probability $p_{sel}^{sel}p_{sel}^{correct}$. It takes c to make the selection.
- The system makes a wrong selection. This happens with probability $p_{sel}^{sel}p_{sel}^{wrong}$. It takes $c+T_{bs}+T_{sel}$ to make the wrong selection, delete the wrong selection and make the intended selection.
- The system decides to skip the trial. This happens with probability $p_{sel}^{skip}$. It takes $c_{T}+T_{sel}$ to skip the trial and make the intended selection.

Then, the expected time for an intended selection is

(15) $$T_{sel}=p_{sel}^{sel}p_{sel}^{correct}c+p_{sel}^{sel}p_{sel}^{wrong}\left(c+T_{bs}+T_{sel}\right)+p_{sel}^{skip}\left(c_{T}+T_{sel}\right).$$

Similarly, the expected time for an intended selection is

(16) $$T_{bs}=p_{sel}^{sel}p_{sel}^{correct}c+p_{sel}^{sel}p_{sel}^{wrong}\left(c+T_{bs}+T_{bs}\right)+p_{sel}^{skip}\left(c_{T}+T_{bs}\right).$$

From (16) and (15), we can find $T_{sel}=T_{bs}$. Then, from (15) we have

(17) $$T_{sel}=T_{bs}=\frac{p_{sel}^{sel}c+\left(1-p_{sel}^{sel}\right)c_{T}}{p_{sel}^{sel}\left(2p_{sel}^{correct}-1\right)}$$

and solve $T_{skip}$ in (14) we get

(18) $$T_{skip}=\frac{p_{skip}^{sel}\left(c+T_{bs}\right)+\left(1-p_{skip}^{sel}\right)c_{T}}{1-p_{skip}^{sel}}\text{,}$$

where we require $p_{sel}^{correct}>0.5$, $p_{sel}^{sel}>0$ and $p_{skip}^{sel}<1$. Then we plug $T_{sel}$ and $T_{skip}$ into (13) to compute the BCI-Utility metric

(19) $$U=\left\{\begin{array}{l} \frac{\pi_{sel}^{i}b_{sel}+\pi_{skip}^{i}b_{skip}}{\pi_{sel}^{i}T_{sel}+\pi_{skip}^{i}T_{skip}},\\ \begin{array}{ll} \text{0,} & \begin{array}{l} p_{sel}^{correct}>0.5\text{,}\;p_{sel}^{sel}>0\;\text{and}\;p_{skip}^{sel}<1\\ p_{sel}^{correct}\leq 0.5\text{,}\;p_{sel}^{sel}=0\;\text{or}\;p_{skip}^{sel}=1 \end{array} \end{array} \end{array}\right.$$

where $T_{sel}$ and $T_{skip}$ are as in (17) and (18), respectively.

We would like to note that the BCI-Utility discussed in previous sections are special cases of the BCI-Utility derived in this section. We have already shown that the Section II-A and Section II-B are special cases of Section II-C. For the case in Section II-C, since there is no intentional trial skip, $\pi_{skip}^{i}=0$ and $\pi_{sel}^{i}=1$ in (13), then (13) reduces to (3). Also, $p_{sel}^{sel}$ and $p_{sel}^{correct}$ in this section would be equivalent to $p^{sel}$ and $p_{sel}^{correct}$ in Section II-C, respectively, because a selection is intended in each trial. Then, (17) reduces to (11), and with the same definition of $b_{sel}$ we reach the same formula for the BCI-Utility in this section and Section II-C.

## III. Simulations and Evaluations

We conduct a series of simulations to evaluate the validity of our BCI-Utility metric under various scenarios. Specifically, we simulate processes that mimic the use of the asynchronous P300 speller using the BCI system described in Section II-D. Note as all previously discussed cases are special cases of Section II-D. For each simulated process, we consider 5,000 intended outcomes, which may include selections or skips, and vary the values of four parameters ($\pi_{sel}^{i}$, $p_{skip}^{sel}$, $p_{sel}^{sel}$, and $p_{sel}^{correct}$) as defined in Section II-D. Possible values of these parameters are summarized in Table I. We select a finer grid in ranges of those parameters that represent BCI systems with desired performance. We consider all combinations across possible values of the four parameters, with a total of 834,176 simulated processes. We set c=14.75 seconds and $c_{T}=31.625$ seconds [29], and $b_{sel}=b_{skip}={\text{log}}_{2}\;\text{(}36\text{)}$ as in a Row-Column Paradigm BCI [3], where N=37 because a trial skip is an additional outcome.

**Fig. 1.**
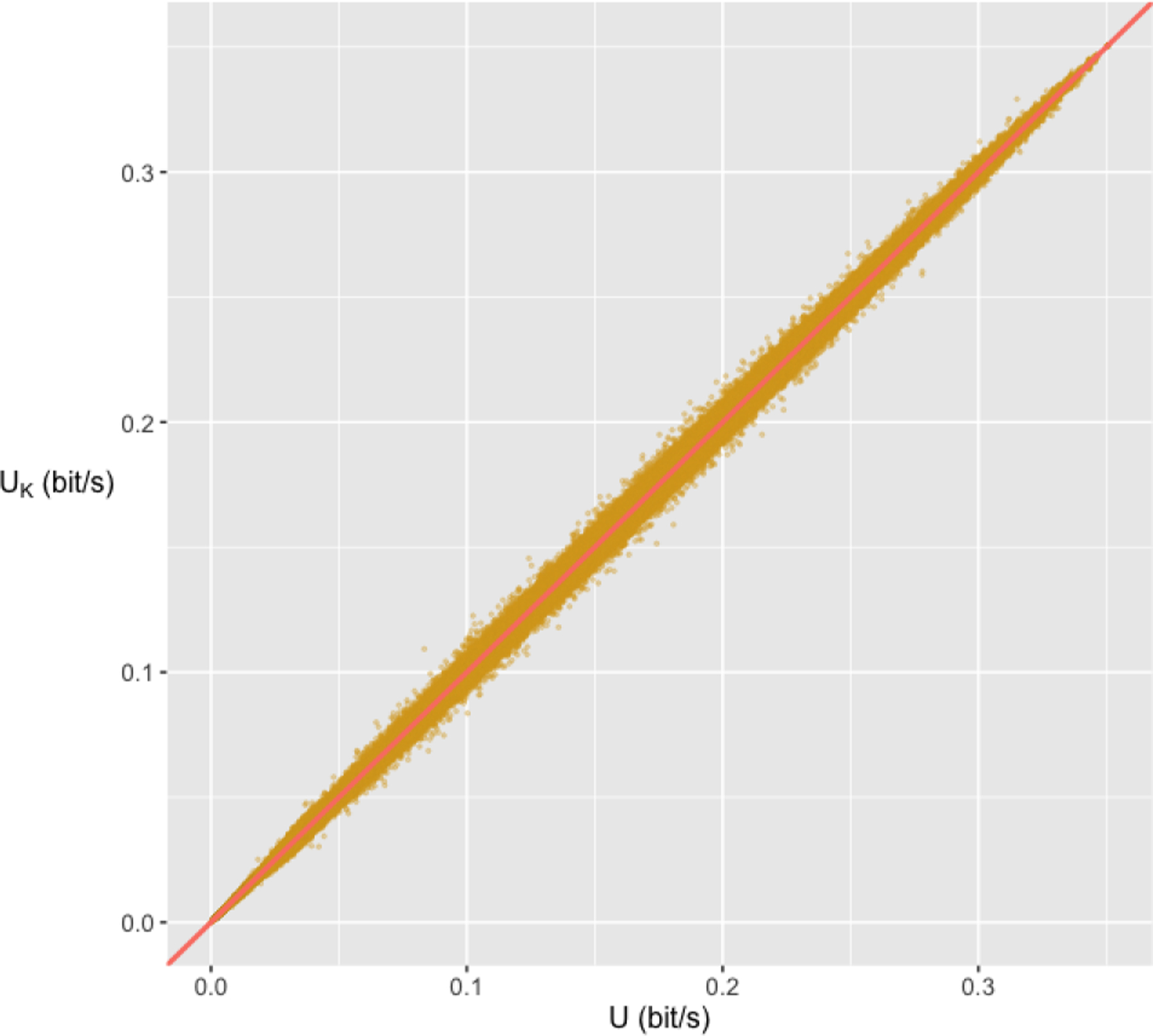
Scatter plot of the observed average benefits in a simulated process $U_{K}$ in bit/s (y-axis) against the BCI-Utility in bit/s computed by our formula U (x-axis). One point is corresponding to one of the 834,176 combinations of parameters.

For each simulated process, we compute the observed average benefits

(20) $$U_{K}=\frac{\sum_{k=1}^{K}b_{k}}{\sum_{k=1}^{K}\text{Δ}t_{k}}$$

where K=5,000 and compute the BCI-Utility U from the formula using the parameters that generate the processes. To evaluate the validity of our BCI-Utility metric, we plot the scatter plot of the {BCI-Utility from formula U, observed average benefits $U_{K}$} pair for all simulated processes (see Figure 1). Each of the 834,176 points corresponds to a simulated process. The points on the scatter plot align well along the diagonal line, indicating that the difference between the observed average benefits and the BCI-Utility computed from our formulas is small across all scenarios, which confirms the validity of our BCI-Utility metric.

To evaluate the variation of the observed average benefits $U_{K}$, we select specific parameter combinations that correspond to desired BCI system performance. For each parameter combination, we simulate 10,000 processes, each consisting of 5,000 intended outcomes, and compute the standard deviation (SD) of $U_{K}$. We present selected parameter combinations and their means and SDs of $U_{K}$ in Table II. We observe that the SD of $U_{K}$ increases as either $\pi_{sel}^{i}$ or $p_{sel}^{sel}$ increases, but remains relatively stable when $p_{slip}^{sel}$ and $p_{sel}^{correct}$ vary. We provide the analysis programs in the supplementary materials to interested readers.

**Fig. 2.**
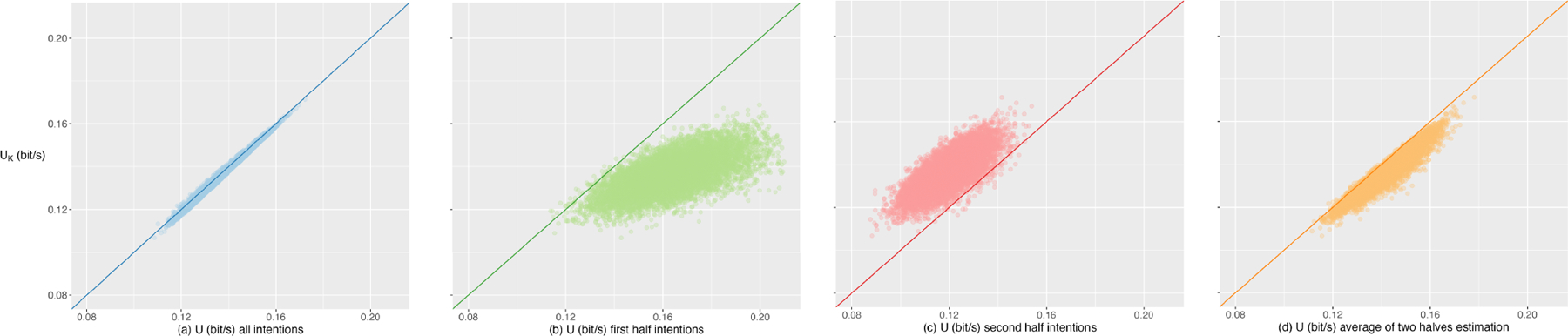
Scatter plots of BCI-Utility U (x-axis, bit/s) versus observed average benefits $U_{K}$ (y-axis, bit/s) for the simulation where assumptions of time-independence of accuracies and memoryless system are violated. We simulate 10,000 processes with violated assumptions. For each simulated process, we compare the observed average benefits $U_{K}$ over all intentions with (a) BCI-Utility for all intentions, (b) BCI-Utility for the first half intentions, (c) BCI-Utility for the second half intentions and (d) the arithmetic mean of the BCI-Utility calculated by the two halves.

We undertake an additional simulation to evaluate the validity of BCI-Utility when the assumptions of time-independent accurary and memoryless system are violated. In practice, when we calculate BCI-Utility by our formula for a specific timeframe of BCI utilization, the proper interpretation is the average benefits of the BCI system over time when the usage time goes to infinity if all critical attributes of the BCI system, including accuracies, the expected time needed for a selection and etc., remain constant in the future. Once the data is recorded, the accuracy within the given duration can be treated as a fixed parameter. Therefore, the BCI-Utility yielded by our formula serves as a reasonably good synopsis of the BCI system’s performance within the observed and fixed usage duration, irrespective of whether the assumptions of time-independence of accuracies and a memoryless system are transgressed. However, the violation of assumptions makes it invalid to generalize the expected average benefits (BCI-Utility) calculated from our formula to the unobserved duration of BCI usage. To elucidate the point, in this simulation, we assume a degradation of accuracies and an increment in the expected time for a selection, both linearly proportionate to the number of intentions. Additionally, we assume that accuracies and the expected time for a selection revert to their initial values for one intention following a mistake. We simulate 10,000 such processes, and each composed of 100 intentions (comprising 20 intended selections succeeded by 5 intended skips, iterated 4 times). The initial accuracy is set to be 90%, and is presumed to decay linearly at a rate of 0.2% per intention. The expected time for a selection is assumed to increase linearly with rate 0.2 second per intention, starting from 14.75 seconds, with a maximum of the complete trial duration $c_{T}=31.625$ seconds. For each simulated process, we calculate the BCI-Utility by (19) for all intentions, the first half of the intentions and the second half of the intentions. We then calculate the average of the first half and second half values. We compare BCI-Utility with the observed average benefit $U_{K}$ calculated over all intentions as in (20) (see Figure 2). Figure 2a shows that the BCI-Utility calculated using all intentions is a accurate estimate of the observed average benefits. As shown in Figure 2b, the BCI-Utility calculated using the first half duration tends to overestimate the observed value, given that it approximates accuracies during a less-decayed duration but we attempt to extrapolate the value to a more-decayed duration. Conversely, computing the BCI-Utility using the second half duration tends to underestimate the observed value due to the estimation of accuracies over a more-decayed duration (Figure 2c). The average benefits calculated by either half exhibit substantial variations. Another observation is that, in this example, the simple arithmetic mean of the BCI-Utility calculated by the two halves does not provide an accurate estimation of the observed average benefits, although it fares better than employing either half in isolation (Figure 2d).

## IV. BCI-Utility Curves Under Different Scenarios

**Fig. 3.**
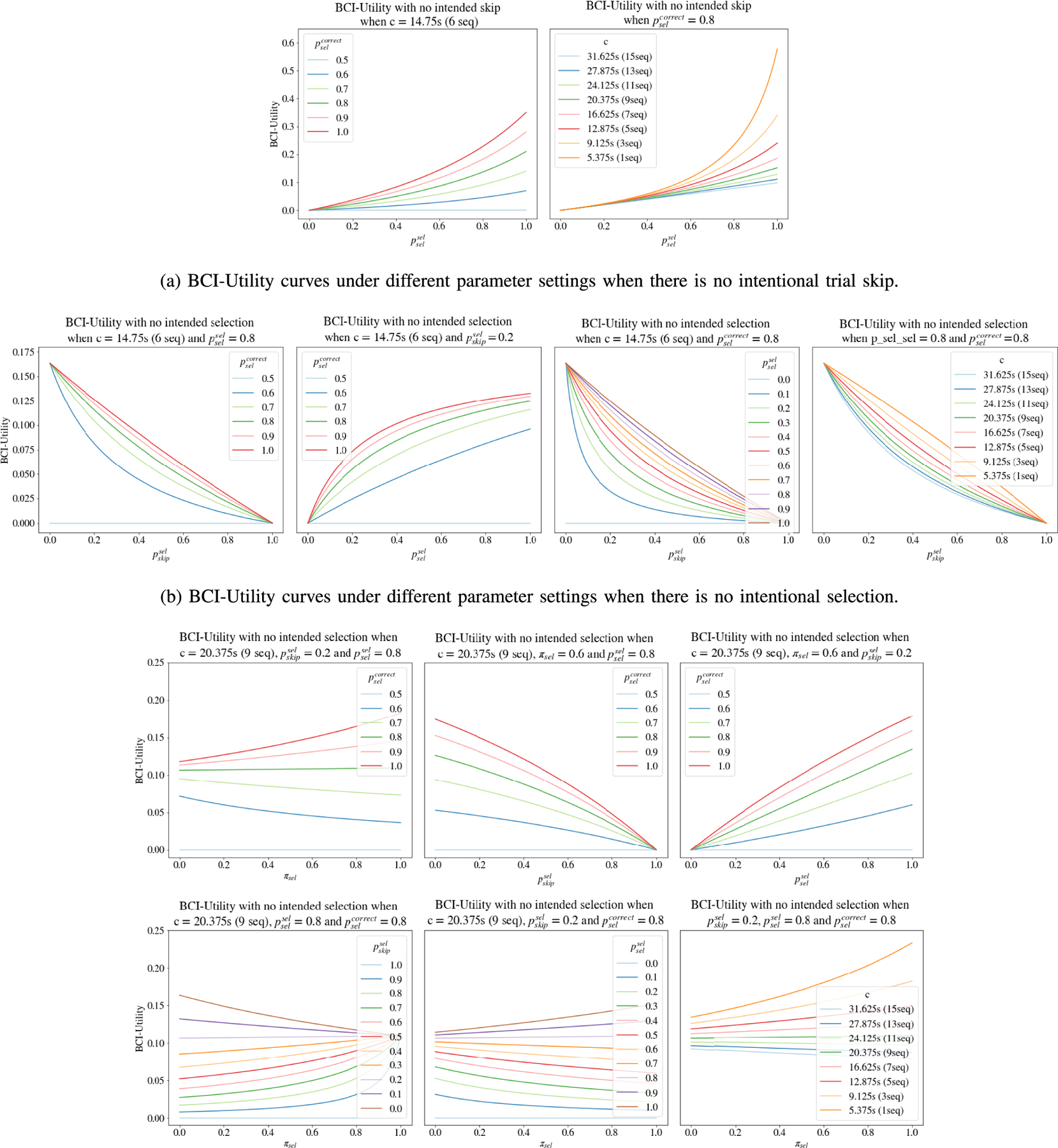
BCI-Utility curves under different parameter settings for three cases: (a) there is no intentional trial skip, i.e., all trials have intended selections; (b) there is no intentional selection, i.e., all trials are intended to be skipped; (c) there are both intentional trial skips and selections. The x-axis represents different parameters; the y-axis always stands for the BCI-Utility value.

To better illustrate the behaviour of the BCI-Utility metric for specific cases, we provide the BCI-Utility curves for a extensive range of parameter settings in Figure 3. We first consider the special cases of continuous typing without any intentional skips (Figure 3a) and of a long period without any intended selections (Figure 3b). Then we consider the general case where both intentional trial skips and selections are possible (Figure 3c). We will use the notations defined in Section II-D, which presents the most general case discussed in this paper. We fix $b_{sel}=b_{skip}={\text{log}}_{2}\;\text{(}36\text{)}$. Generally, the value of the BCI-Utility metric increases as the accuracy increases, decreases as the error rate increases, and decreases as the expected time c for an intended selection increases. In our discussion, we define accuracy and error rate in a broad sense. We consider accuracy as any of the following three – the probability of a correct selection given that a selection is intended and made $\text{(}p_{sel}^{correct}\text{)}$, the probability of the system making a selection given a selection is intended $\text{(}p_{sel}^{sel}\text{)}$, and the probability of the system skipping a trial given a trial skip is intended $\text{(}p_{skip}^{skip}\text{)}$. Similarly, the error rate includes $p_{sel}^{wrong}=1-p_{sel}^{correct}$, $p_{sel}^{skip}=1-p_{sel}^{sel}$ and $p_{skip}^{sel}=1-p_{skip}^{skip}$.

The first case (Figure 3a) is when there is no intentional trial skip (i.e., all intended outcomes are selections), thus $\pi_{skip}^{i}=0$ in (13). This corresponds to the scenario discussed in Section II-C where a long period of continuous use is expected, and only three parameters are involved ($p_{sel}^{sel}$, $p_{sel}^{correct}$ and c). The BCI-Utility metric has an increasing trend as the probability of the system making a selection $\text{(}p_{sel}^{sel}\text{)}$ increases (in this case, as all intended outcomes are selections, $p_{sel}^{sel}$ has the same meaning of $p^{sel}$ in Section II-C). A higher selection accuracy $\text{(}p_{sel}^{correct}\text{)}$ and a shorter expected time for an intended selection lead to a higher BCI-Utility.

The second case (Figure 3b) is when there is no intentional selection (i.e., all intended outcomes are trial skips), which corresponds to the scenario where a long period of no-control is expected. In this case, $\pi_{sel}^{i}=0$, and (13) reduces to $U=b_{skip}\text{/}T_{skip}$. However, even if all intended outcomes are trial skips, $p_{sel}^{sel}$ and $p_{sel}^{correct}$ are involved as in (17) and (18) because when the system mistakenly makes a selection, the user would want to type a “backspace” to delete the unintended character. Therefore, four parameters are involved ($p_{sel}^{sel}$, $p_{sel}^{correct}$, $p_{skip}^{sel}$ and c). The BCI-Utility metric increases as the selection accuracy $\text{(}p_{sel}^{correct}\text{)}$ or the probability of the system making a selection given that a selection is intended $\text{(}p_{sel}^{sel}\text{)}$ increases, and decreases as the probability of the system making a selection given that a skip is intended $\text{(}p_{skip}^{sel}\text{)}$ increases. A shorter expected time for an intended selection yields a higher BCI-Utility. Note that the time for an intended skip is always fixed at $c_{T}$.

The third case (Figure 3c) is more general in that both intentional trial skips and selections are possible. Five parameters are involved in the calculation of the BCI-Utility metric ($\pi_{sel}^{i}$, $p_{sel}^{sel}$, $p_{sel}^{correct}$, $p_{skip}^{sel}$ and c). The trend of the BCI-Utility metric is similar as previously discussed – increases as accuracy increases or the expected time of an intended selection decreases, and decreases as error rate increases. However, the BCI-Utility may either increase or decrease as $\pi_{sel}^{i}$ varies, depending on other parameters. This inconsistency is expected. The BCI-Utility metric is in favor of a higher probability of intended selections $\text{(}\pi_{sel}^{i}\text{)}$ in situations with high $p_{sel}^{correct}$ and $p_{sel}^{sel}$. In contrast, the BCI-Utility is higher when $\pi_{sel}^{i}$ decreases in the case where $p_{skip}^{skip}=1-p_{skip}^{sel}$ is relatively high. However, fixing all other parameters, a higher accuracy would always lead to a higher BCI-Utility.

Besides the general trend, there are more implications from the BCI-Utility curves. First, the BCI-Utility metric generally does not change linearly as a parameter changes. In many situations, changing one parameter is more effective than changing other parameters for obtaining a higher BCI-Utility. In addition, the effect of $\pi_{sel}^{i}$ on the BCI-Utility metric is highly dependent on other parameters. Therefore, to optimize the system performance in terms of the BCI-Utility metric, we suggest researchers take partial derivatives of the BCI-Utility formula with respect to all parameters, and then determine the most effective parameter to optimize to improve performance by looking for the most sensitive parameters at the point of current system performance. Finally, in many cases, shortening the expected time of an intended selection is the most effective way to improve the BCI-Utility. This necessitates further development of asynchronous BCI systems capable of abstention and dynamic stopping.

## V. Discussion of Application to Real Data

We apply the derived BCI-Utility metric to real-world BCI performance data, thereby illustrating the practical use of this metric. For this example, we use data collected from a single research participant who gave informed consent to a protocol approved by the University of Michigan Institutional Review Board. The dataset comprises 117 target outcomes, with 107 intended selections and 10 intended skips. The keyboard had either 49, 55, 63, or 69 possible selections and each target outcome had three sequences of recorded data with 20 or 24 stimulus groups per sequence depending on the number of possible selections. Stimuli were presented for 125 ms with 62.5 ms between stimuli, so each sequence was either 3750 ms or 4500 ms in duration. There were 10,000 milliseconds between selections.

The data was subjected to analysis using an offline dynamic stopping and abstention algorithm. The details of the algorithm are irrelevant to the application of the metric. The calculation of the BCI-Utility relies on several parameters extracted from the performance data. Firstly, accuracies are directly estimated as per their definition. The probability of the system making a selection when a selection is intended $\text{(}p_{sel}^{sel}\text{)}$ is the percentage of the number of intended selections where a selection was produced. For the example dataset, there were 107 intended selections and 94 of these resulted in a selection being made, so $p_{sel}^{sel}=87.\text{9\%}$. Likewise, the dataset had 10 intended skips and 8 were correctly identified, so $p_{skip}^{skip}=80.\text{0\%}$. For $p_{sel}^{correct}$, it is important to note that this is the percentage of correct selections that resulted when a selection was intended. So, although the BCI made 96 selections, only 94 were made when a selection was intended. Thus $p_{sel}^{correct}=75\text{/}94=80.0\text{\%}$. Then, we approximate the proportions of intended selections by $\;\pi_{sel}^{i}=107\text{/}117=91.5\text{\%}$.

For this dataset, the maximum trial length is 3 sequences. If all sequences were of uniform length, then the expected time for a complete trial $c_{T}$ would be the number of sequences in a complete trial times the length of a sequence plus the time between selections. Since our data has variations in the length of a sequence, we estimate the expected time for a complete trial $c_{T}$ by averaging the duration of all complete trials then adding the 10-second delay between selections. Since there are 16 trials with duration of 3750 ms and 101 trials with 4500 ms, $c_{T}=\frac{16\times 3750+101\times 4500}{117}+10\text{,}000=23192\;\text{ms}$, which is 23.19 seconds.

The expected time c for a selection is approximated as the average time taken for any selection. Again, if the sequence length were constant, this could be calculated by multiplying the average number of sequences (1.64 in our example) times the length of a sequence and adding the time between selections. However, for our data with variable sequence duration for different screens, we take the average duration of all the selections (7.21 seconds) and add the 10-second delay between selections to get c=17.21 seconds per selection.

The benefit for a selection and the benefit for a skip is user-specified. One may choose the unit benefit, which gives the BCI-Utility an interpretation of selection rate, while here, we utilize $b_{sel}=b_{skip}={\text{log}}_{2}\text{(}N\text{)}$, with N signifying the number of on-screen keys, thus attributing an information transfer rate interpretation to the BCI-Utility. When the count of on-screen keys fluctuates across different trials, we suggest calculating average benefits through averaging ${\text{log}}_{2}\text{(}N\text{)}$ across trials. For this dataset, $b_{sel}=b_{skip}=5.8$.

With all the parameters estimated from data, we first compute $T_{sel}=34.27$ seconds according to (17) and $T_{skip}=36.06$ seconds according to (18). Then, the BCI-Utility for this duration of BCI usage is 0.169 bit/s by plugging needed values into (19), effectively measuring system throughput by amalgamating accuracy, typing speed, and the benefit of intentional periods without typing.

However, computing the proportion of intentional selections $\text{(}\pi_{sel}^{i}\text{)}$ is more intricate than a straightforward ratio of intentional selections to total trials. It is noteworthy that, in our derivation, intentional selections made for error correction purposes do not yield benefits and are considered part of the fulfillment of initially intended selections. For instance, if a user intends to type a character but the system erroneously selects another, subsequent actions like utilizing backspace to rectify the error and then typing the correct character do not contribute to the proportion of intentional selections due to the absence of benefits. In essence, the proportion of intentional selections encompasses solely the initially intended selections, omitting error-correction selections. While determining initially intended selections is relatively uncomplicated in online data, it poses challenges in offline scenarios in which classification, dynamic stopping, or abstention algorithms are applied and compared, since using recorded data will evaluate an algorithm based on data that would not have been generated by that algorithm if used online.

Nonetheless, in practical terms, we can still compute the proportions of intended selections and skips on a per-selection basis, incorporating the intentional selections for error correction. In most cases, because the ratio of error correction to total selections is small, this would only slightly shift the estimated proportion of intended selections from its true value if corrections are executed. Additionally, it leaves other parameters unaffected. These deviations in $\pi_{sel}^{i}$ would have small impact on the computed BCI-Utility, particularly when other parameters fall within reasonable ranges, as demonstrated in Figure 3. From Figure 3c, as $\pi_{sel}^{i}$ varies, the BCI-Utility values remain stable, so even with the inclusion of error correction selections that would not be needed by a better-preforming algorithm, our BCI-Utility formula still serves as an reasonable approximation.

## VI. Conclusion

In this work, we present the BCI-Utility metric for evaluating asynchronous P300 spellers. We consider three asynchronous cases – dynamic stopping, dynamic stopping and abstention, and finally dynamic stopping with abstention and intentional idle periods. While our work is in the context of the P300 speller, the derived metric should also be applicable to other asynchronous BCIs.

The derived BCI-Utility metric is particularly useful for dynamic stopping and abstention algorithm comparison on offline recorded data in which the trial duration is fixed at the time of recording. We assume time-independence for accuracy, error rate and the proportion of intended selection to simplify the derivations. However, in a long period of BCI use, users may get tired, which is likely to result in a lower accuracy and a higher error rate. The proportion of intended selections may also vary over time with periods of active use and periods of relatively little use.

Online use of an asynchronous BCI may most fully be implemented by making decisions based on the EEG responses within a sliding window of a fixed or variable number of sequences. In this design, two consecutive sliding windows are dependent because they only differ by the first and last sequence and overlap in the middle sequences. Thus, the time-independent accuracy and memoryless system assumptions required in our derivations are violated. Therefore, future investigations are needed to develop a BCI-Utility metric that can be applied to the sliding window BCI design. However, it may be worth noting that once recorded, data from a sliding window design can be evaluated using the currently derived BCI-Utility formulas by considering the data preceding each selection as a separate trial with a fixed number of sequences.

## Supplementary Material

tnsre-3322125-mm

## Figures and Tables

**TABLE I:** Possible Values of the Parameters in the Simulations. A Total of 38 × 28 × 28 × 28=834,176 Cases are Considered

Parameters	Possible Values	# Values
$\pi_{sel}^{i}$	0 to 0.7 step 0.1 and 0.71 to 1 step 0.01	38
$p_{skip}^{sel}$	0 to 0.19 step 0.01 and 0.2 to 0.9 step 0.1	28
$p_{sel}^{sel}$	0.1 to 0.8 step 0.1 and 0.81 to 1 step 0.01	28
$p_{sel}^{correct}$	0.55 to 0.75 step 0.1 and 0.76 to 1 step 0.01	28

**TABLE II:** The Parameter Combinations ($\pi_{sel}^{i}$, $p_{skip}^{sel}$, $p_{sel}^{sel}$ and $p_{sel}^{correct}$) and the SD ($\times 10^{-3}$ BIT/S) and Mean ($\times 10^{-3}$ BIT/S) of Observed Average Utility Estimated Over 10,000 Simulated Processes

Parameters				SD $\text{(}\times 10^{-3}\text{)}$	Mean $\text{(}\times 10^{-3}\text{)}$
$\pi_{sel}^{i}$	$p_{skip}^{sel}$	$p_{sel}^{sel}$	$p_{sel}^{correct}$		
0.75	0.15	0.85	0.85	2.13	163.52
0.80				2.22	166.22
0.85				2.29	169.02
0.90				2.40	171.92
0.95				2.50	174.93
0.85	0.05	0.85	0.85	2.32	173.66
	0.10			2.34	171.43
	0.15			2.29	169.02
	0.20			2.29	166.40
	0.25			2.31	163.49
0.85	0.15	0.75	0.85	2.08	140.49
		0.80		2.22	154.33
		0.85		2.29	169.02
		0.90		2.43	184.63
		0.95		2.44	201.28
0.85	0.15	0.85	0.75	2.25	126.80
			0.80	2.29	148.42
			0.85	2.29	169.02
			0.90	2.30	188.67
			0.95	2.26	207.34
