# Supplementary material for: BCI-Utility Metric for Asynchronous P300 Brain-Computer Interface Systems
Source: IEEE Trans Neural Syst Rehabil Eng. Author manuscript; Available in PMC 2023 Nov 27. (PMC10681042; doi:10.1109/TNSRE.2023.3322125)
Supplement: tnsre-3322125-mm [file NIHMS1938193-supplement-tnsre-3322125-mm.zip › tnsre-3322125-mm/supplementary materials description.pdf]

Description of Supplementary Materials

This is a description of supplementary materials of *BCI-Utility Metric for Asynchronous P300 Brain-Computer Interface Systems*. Please cite:

Ma, G., Kang, J., Thompson, D. E., & Huggins, J. E. (2023). BCI-Utility Metric for Asynchronous P300 Brain-Computer Interface Systems. *IEEE transactions on neural systems and rehabilitation engineering.*

Dal Seno, B., Matteucci, M., & Mainardi, L. T. (2009). The utility metric: a novel method to assess the overall performance of discrete brain–computer interfaces. *IEEE Transactions on Neural Systems and Rehabilitation Engineering*, 18(1), 20-28.

Software versions: Matlab R2022a, python 3.9, R 4.2.0

We provide three functions to calculate our derived utility. Please refer to Section II of the paper for the derived formula.

1. `compute_BCI_utility.m`: Matlab code for computing utility from predicted outcomes and intended outcomes
2. `compute_BCI_utility_from_prob.py`: python code for computing utility using estimated probabilities
3. `compute_BCI_utility_from_prob.R`: R code for computing utility using estimated probabilities

We also provide code for generating the Figure 1, Figure 2, Figure 3 and Table 2. Table 1 is a description of parameter combinations. Please refer to Section III of the paper for the detailed description of simulations.

1. `figure1_utility_simulation.R`: code for simulating 834,176 processes of different parameter combinations (described in Table 1) and generating Figure 1
2. `figure2_misassumption_simulation.R`: simulation code for violated assumptions, Figure 2 can be reproduced by this file
3. `figure3.ipynb`: Jupyter notebook (python) for plotting Figure 3
4. `table2_zoom_in_typical.R`: code for simulating 10,000 processes for each of the selected parameter combinations and generating numbers in Table 2
5. `usimu.R`: helper functions for simulations
6. `simulation.rdata.rdata`: saved results for the 834,176 simulated processes
7. `zoom_in_typical.rdata`: saved results for the 10,000 simulated processes for each of the selected parameter combinations

If readers are interested into doing more simulations, we provide two functions in `umisu.R` for the purpose. Please refer to Section III of the paper for the detailed description of simulations.

1. `simulation` is a function that runs simulations for all combinations of provided probability parameters, one simulated process for each parameter setting. An example of using the code can be found in `figure1_utility_simulation.R`.
2. `zoom_in_simulation` is a function that simulates multiple processes for each of the given parameter combinations, and one can use the results to evaluate the variation of the observed average benefits. An example of using the function can be found in `table2_zoom_in_typical.R`.
